# The Effect of Glucagon-Like Peptide 1 Receptor Agonists on Weight Loss in Type 2 Diabetes: A Systematic Review and Mixed Treatment Comparison Meta-Analysis

**DOI:** 10.1371/journal.pone.0126769

**Published:** 2015-06-29

**Authors:** Jessica E. Potts, Laura J. Gray, Emer M. Brady, Kamlesh Khunti, Melanie J. Davies, Danielle H. Bodicoat

**Affiliations:** 1 University of Leicester, Department of Health Sciences, Leicester, United Kingdom; 2 Leicester Diabetes Centre, University Hospitals of Leicester NHS Trust, Leicester, United Kingdom; 3 University of Leicester, Diabetes Research Centre, Leicester, United Kingdom; Weill Cornell Medical College Qatar, QATAR

## Abstract

**Aims:**

To determine the effects of glucagon-like peptide-1 receptor agonists compared with placebo and other anti-diabetic agents on weight loss in overweight or obese patients with type 2 diabetes mellitus.

**Methods:**

Electronic searches were conducted for randomised controlled trials that compared a glucagon-like peptide-1 receptor agonist therapy at a clinically relevant dose with a comparator treatment (other type 2 diabetes treatment or placebo) in adults with type 2 diabetes and a mean body mass index ≥ 25kg/m^2^. Pair-wise meta-analyses and mixed treatment comparisons were conducted to examine the difference in weight change at six months between the glucagon-like peptide-1 receptor agonists and each comparator.

**Results:**

In the mixed treatment comparison (27 trials), the glucagon-like peptide-1 receptor agonists were the most successful in terms of weight loss; exenatide 2mg/week: -1.62kg (95% CrI: -2.95kg, -0.30kg), exenatide 20μg: -1.37kg (95% CI: -222kg, -0.52kg), liraglutide 1.2mg: -1.01kg (95%CrI: -2.41kg, 0.38kg) and liraglutide 1.8mg: -1.51 kg (95% CI: -2.67kg, -0.37kg) compared with placebo. There were no differences between the GLP-1 receptor agonists in terms of weight loss.

**Conclusions:**

This review provides evidence that glucagon-like peptide-1 receptor agonist therapies are associated with weight loss in overweight or obese patients with type 2 diabetes with no difference in weight loss seen between the different types of GLP-1 receptor agonists assessed.

## Introduction

The World Health Organisation estimates that over 1.4 billion adults were overweight in 2008, and of these 500 million were obese [1]. Obesity (defined as a body mass index ≥30kg/m^2^) increases the risk of developing type 2 diabetes mellitus, a condition where blood glucose levels are elevated due to decreased insulin production and/or sensitivity. It is estimated that there are 347 million people with diabetes worldwide [2]; type 2 diabetes accounts for between 85–95% of these cases. The relationship between obesity and increased risk of major complications in type 2 diabetes, including mortality is well documented [3] and concerning given the current increasing rates of obesity. Weight reduction is a key intervention for people with type 2 diabetes [4]. When diet and lifestyle modifications have not elicited improvements in glycaemic control the first-line treatment for type 2 diabetes is metformin, with further therapies being added as necessary, including sulfonylureas, thiazolidinediones, GLP-1 receptor agonists and DPP-IV inhibitors [4]. Unfortunately, not all of these therapies are weight neutral and some can lead to weight gain [5]. A review of GLP-1 receptor agonists showed that these can lead to weight loss in obese or overweight patients with type 2 diabetes [6]. Exenatide, liraglutide and lixisenatide are GLP-1 receptor agonists that are currently used treatments for overweight patients with type 2 diabetes.

A traditional pair-wise meta-analysis has been conducted in this area, comparing data from two GLP-1 receptor agonists combined (exenatide and liraglutide) against a control [6]. Grouping exenatide and liraglutide may not be the best approach as they are different drugs; for example, exenatide has a 50% amino acid homology to GLP-1 whereas liraglutide has a 97% homology and thus a longer half-life. Furthermore, they are administered with different frequencies. Therefore, they may have different effects on weight loss. In order to examine the different effects of the GLP-1 receptor agonist therapies on weight loss, a mixed treatment comparison meta-analysis was performed to estimate the treatment effects of each intervention individually. Mixed treatment comparison meta-analyses allow direct and indirect evidence to be combined, allowing treatment comparisons where no head-to-head trials exist through a common comparator.

## Materials and Methods

### Literature search and inclusion criteria

We identified publications published up to June 2013 from searches of Medline and Embase. The search strategy used free text terms and keywords to identify randomised controlled trials assessing GLP-1 receptor agonist therapies that reported a weight change (an example search strategy is given in S1 File). The titles and abstracts of all studies identified by the electronic searches were screened for inclusion by one reviewer (JP). The full texts of all studies found to be potentially relevant were assessed by three individuals (JP, DB and LG).

We included studies meeting the following inclusion criteria: 1) randomised controlled trial, 2) published in English language, 3) adult participants (age ≥ 16 years) with type 2 diabetes, 4) mean body mass index of all participants in the study ≥ 25kg/m^2^, 5) at least one licensed GLP-1 receptor agonist therapy treatment arm administered for 6 months at a dose given in clinical practice in the UK, and 6) weight reported as an outcome at six months, as this was the most common time reported. Studies in adults without type 2 diabetes were excluded since there were too few studies in that population to allow for a meaningful evidence synthesis. There were no restrictions placed on the treatment given to the control group. Neither were restrictions placed on any other oral anti-diabetic treatments the participants may already be receiving. Studies comparing two different GLP-1 receptor agonist therapies were also included. The reference lists of pooled or secondary analyses were hand searched for papers that were eligible for inclusion, although no additional papers were identified through this method.

### Data extraction and quality assessment

Data extraction was performed on the full texts that were eligible for inclusion by three individuals (JP, DB, and LG), who used a standard data extraction template. Any issues found with the information that studies reported were discussed and checked by a second individual. The following data were extracted: author, year of publication, journal of publication, study country, number of treatment arms, type of treatment, treatment duration, interventions received, time points when data were collected, number of withdrawals, and number lost to follow-up, as well as mean body mass index, mean age and percentage of female participants for each treatment arm. Information was also extracted on the mean weight (kg) and standard deviation (SD) at baseline and 6 months (we allowed the mean follow up to vary between 4–8 months post randomisation), and/or the change in mean weight and SD of each treatment arm, whichever was reported in the trial. Some papers also reported the difference between the treatment arms which was extracted if arm level data were not reported. The quality of the studies was assessed by looking at the main areas in which bias can occur in the study: randomisation of the treatments, allocation concealment, blinding of the study, and the flow of participants.

### Statistical analysis

For studies that had given clinically relevant doses of the treatments for six months, a pair-wise meta-analysis was carried out followed by a mixed treatment comparison meta-analysis. As all patients had type 2 diabetes it was assumed that they would all be receiving similar background treatments and so the interventions were simplified by ignoring the background treatments. As well as this, the dose for the non GLP-1 receptor agonist treatments was ignored. Where SDs for means were not reported, these were estimated from standard errors, ranges, p-values or 95% confidence intervals as appropriate [7]. Where data on baseline and follow-up weight were reported, the mean change was calculated and the SD was imputed [7]. Any SD less than one was assumed to have been misreported and assumed to be a standard error (n = 4 [8–11]).

The pair-wise meta-analyses pooled studies in Stata Version 12 using a random effects model as studies were expected to be heterogeneous. Heterogeneity was assessed using the I^2^ statistic. Publication bias was assessed visually using contour enhanced funnel plots [12] for all comparisons that contained five or more studies.

Mixed treatment comparison methods were used to compare all interventions under investigation within a single model. There was a continuous outcome of mean change in body weight from baseline between treatments. Placebo was used as the reference category throughout. A random effects mixed treatment comparison meta-analysis was conducted using a linear regression model adjusting for the fact that some trials had more than two treatment arms [13]. The model was fitted with a burn in of 10,000 samples which were discarded, followed by 20,000 samples that were recorded. For each treatment, the percentage of times that the treatment gained the highest rank across all of the simulations was calculated. Sensitivity to the length of burn in, sample size and initial values was examined. Convergence of the initial values was checked by Brooks-Gelman-Rubin plots [14]. History plots were used to assess whether the length of burn in and sample size were adequate. The residual deviance was used to assess model fit with the residual deviance for a good fitting model lying around the number of unconstrained data points. All mixed treatment comparison analyses were conducted using a Markov chain Monte Carlo simulation method in WinBUGS 1.4.3, with vague prior distributions.

The results of the pair-wise meta-analysis and the mixed treatment comparison were compared for inconsistencies in the mean differences calculated by each method. The results were defined to be inconsistent if the estimate calculated by the mixed treatment comparison did not fall in the 95% confidence interval calculated from the pair-wise analysis.

## Results

### Study Characteristics

**Fig 1:**
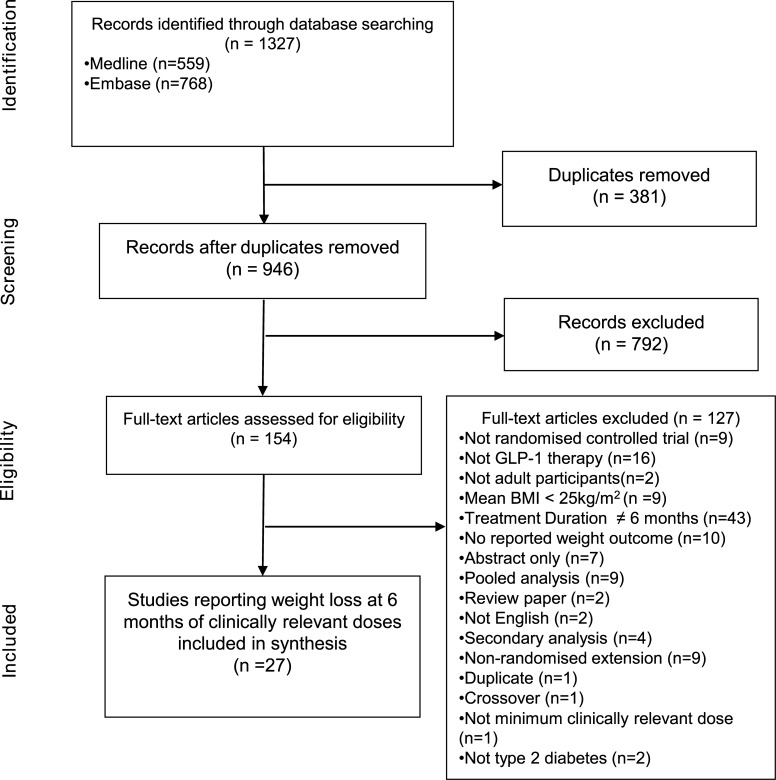
PRISMA flow diagram of study selection.

Searches identified 1327 records from the electronic databases for inclusion in the systematic review (Fig 1). After removing 381 duplicate records, 946 titles and abstracts were screened. The initial screening process found that 792 records were not suitable for inclusion. Full texts were assessed for the remaining 154 records. There were 127 records excluded leaving 27 studies that fulfilled the inclusion criteria and had data extracted for inclusion in the meta-analyses (Fig 1).

**Fig 2:**
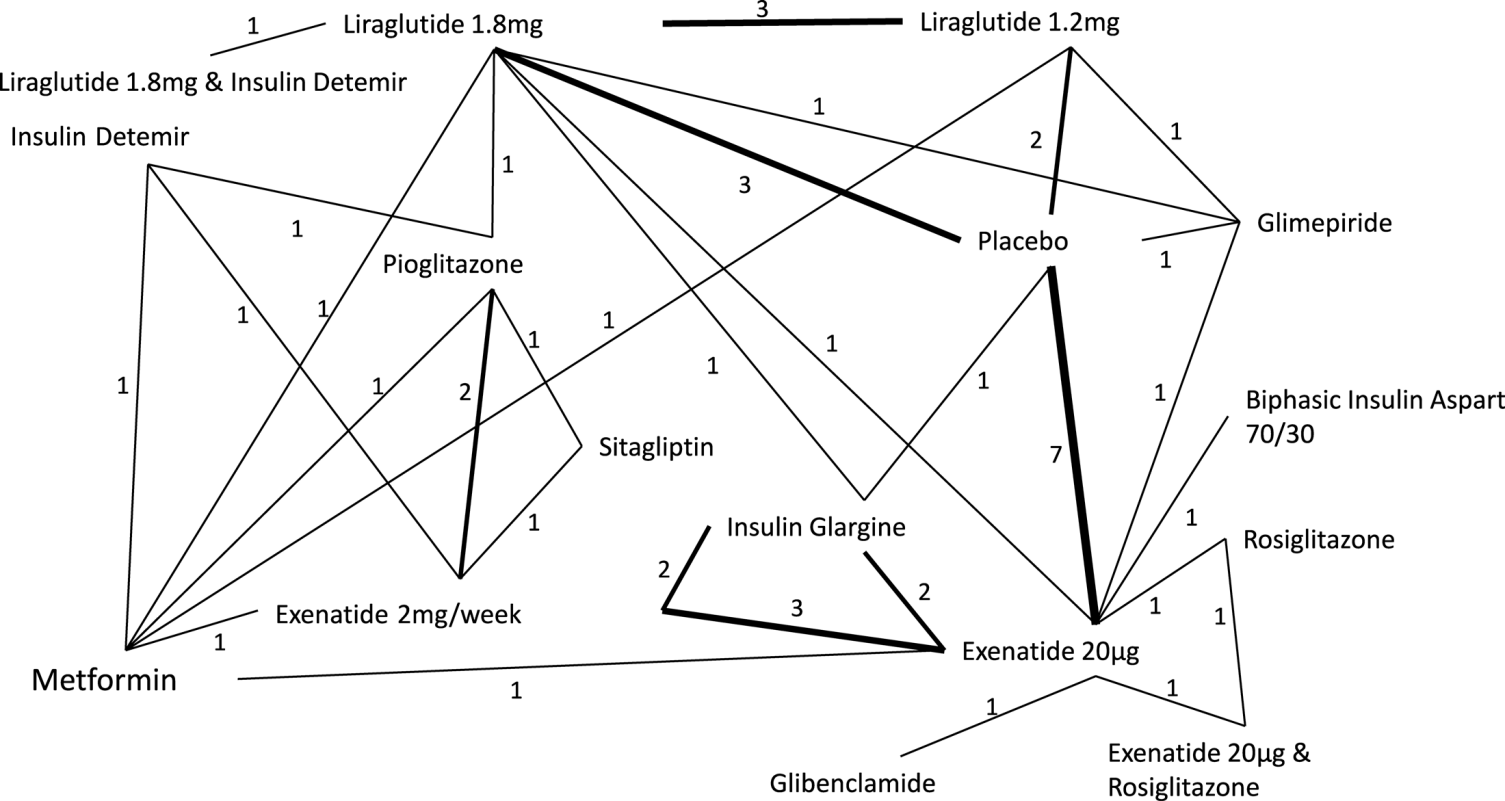
Network diagram of treatments comparisons in analysis. Numbers represent the number of studies that reported a direct comparison between each pair of treatments. Line thickness is weighted so that a thicker line represents a higher number of direct comparisons.

In the 27 trials, 16 different interventions were given, and there were 31 direct comparisons reported between the different interventions (Fig 2). The GLP-1 receptor agonist therapies included were exenatide 20μg daily, exenatide 2mg/week, liraglutide 1.2mg daily and liraglutide 1.8mg daily. Lixisenatide was not included because no eligible studies considering this treatment were identified in the review process. Control interventions included placebo and metformin among others (Fig 2). According to the inclusion criteria, all included trials reported a mean body mass index of > 25kg/m^2^ (range 25.8kg/m^2^–35.0kg/m^2^). The age of participants ranged from 51 years to 60 years. The minimum percentage of females included was 29.7% ranging up to trials where 63% of the participants were female (Table 1).

**Table 1:** Summary of included trials.

Author (year)	Interventions	Country	Number of participants	Treatment Duration (months)	Mean BMI (kg/m2)	Mean Age (years	Females (%)	Background Treatment
Apovian (2010) [23]	Exenatide 20μg	USA	96	5.5	33.6	54.5	63	Pharmaceutical
	Placebo		98	5.5	33.9	55.1	62	Pharmaceutical
Bergenstal (2009) [24]	Exenatide 20μg	USA	124	5.5	34.2	52.5	51.6	Pharmaceutical
	Biphasic Insulin aspart		248	5.5	33.6	52.4	52	Pharmaceutical
Bergenstal (2010) [25]	Exenatide 2mg/week	USA, India, Mexico	170	6	32	52	44	Pharmaceutical
	Sitagliptin 100mg		172	6	32	52	48	Pharmaceutical
	Pioglitazone 45mg		172	6	32	53	52	Pharmaceutical
Blevins (2011) [8]	Exenatide 20μg	USA	123	5.5	33	55	45	Pharmaceutical
	Exenatide 2mg/week		129	5.5	33.6	56	40	Pharmaceutical
Buse (2011) [26]	Exenatide 20μg	Greece, Israel, Mexico,	137	6.9	33.8	59	49	Pharmaceutical
	Placebo	UK, USA	122	6.9	33.1	59	36	Pharmaceutical
Buse (2004) [9]	Exenatide 20μg	USA	125	6.9	33	56	42.6	Pharmaceutical
	Placebo		123	6.9	34	55	37.4	Pharmaceutical
Buse (2009) [43]	Exenatide 20μg	Austria, Denmark, Finland, France, Germany, Ireland, Macedonia, Norway, Poland, Puerto Rico, Romania, Slovenia, Spain, Sweden, Switzerland, USA	231	6	32.9	57.1	45	Pharmaceutical
	Liraglutide 1.8mg		233	6	32.9	56.3	51	Pharmaceutical
Davies (2013) [27]	Exenatide 2mg/week	UK	111	6	33.7	59	36	Pharmaceutical
	Insulin detemir		105	6	33.7	58	31	Pharmaceutical
Davies (2009) [28]	Exenatide 20μg	UK	118	6	34.6	56.8	29.7	Pharmaceutical
	Insulin glargine		117	6	33.7	56.2	33.6	Pharmaceutical
DeFronzo (2010) [29]	Exenatide 20μg	USA	45	4.6	32.5	56	49	Non- Pharmaceutical
	Exenatide 20μg + Rosiglitazone 8mg		47	4.6	32.5	56	49	Non- Pharmaceutical
	Rosiglitazone 8mg		45	4.6	32.5	56	49	Non- Pharmaceutical
DeFronzo (2005) [30]	Placebo	USA	113	6.9	34	54	40.7	Pharmaceutical
	Exenatide 20μg		113	6.9	34	52	39.8	Pharmaceutical
Derosa (2010) [31]	Exenatide 20μg	Italy	63	6	28.7	57	52.4	Pharmaceutical
	Glibenclamide 15mg		65	6	28.5	56	49.2	Pharmaceutical
Derosa (2011) [32]	Exenatide 20μg	Italy	57	6	28.4	56	50.9	Pharmaceutical
	Glimepiride 6mg		54	6	28.5	55	51.9	Pharmaceutical
DeVries (2011) [44]	Insulin Detemir + Liraglutide 1.8mg	Belgium, Canada, France, Germany, Italy, Netherlands, Spain, UK, USA	162	6	34.9	56.8	45.7	Pharmaceutical
	Liraglutide 1.8mg		161	6	33.9	57.3	44.7	Pharmaceutical
Diamant (2010) [33]	Exenatide 2mg/week	USA, Puerto Rico, European Union, Russia, Australia, Republic of Korea, Taiwan, Mexico	233	6	32	58	48	Pharmaceutical
	Insulin glargine		223	6	32	58	45	Pharmaceutical
Drucker (2008) [34]	Exenatide 2mg/week	USA, Canada	148	7.5	35	55	45	Pharmaceutical
	Exenatide 20μg		147	7.5	35	55	49	Pharmaceutical
Heine (2005) [35]	Exenatide 20μg	Australia, Belgium, Brazil, Finland, USA, Germany, Netherlands, Poland, Norway Puerto Rico, Portugal, Spain, Sweden	282	6	31.4	59.8	45	Pharmaceutical
	Insulin glargine		267	6	31.3	58	43.4	Pharmaceutical
Ji (2013) [11]	Exenatide 2mg/week	China, India, Japan, South Korea, Taiwan	340	6	26.4	55	46.2	Pharmaceutical
	Exenatide 20μg		338	6	26.7	56	45.6	Pharmaceutical
Kadowaki (2011) [36]	Placebo	Japan	36	6	25.8	56.3	31.4	Pharmaceutical
	Exenatide 20μg		73	6	25.8	59.4	31.9	Pharmaceutical
Kendall (2005) [37]	Placebo	USA	247	7.5	34	56	44.1	Pharmaceutical
	Exenatide 20μg		241	7.5	34	55	40.7	Pharmaceutical
Liutkus (2010) [38]	Exenatide 20μg	Canada, Mexico, Romania, South Africa, USA	111	6	34	55	40	Pharmaceutical
	Placebo		54	6	33	54	43	Pharmaceutical
Nauck (2009) [10]	Liraglutide 1.2mg	Argentina, Australia, Belgium, Bulgaria, Croatia, Denmark, Germany, Hungary, India, Ireland, Italy, Netherlands, New Zealand, Norway, Romania, Russia, Slovakia, South Africa, Spain, Sweden, UK	240	24	31.1	57	46	Pharmaceutical
	Liraglutide 1.8mg		242	6	30.9	57	46	Pharmaceutical
	Glimepiride 4mg		242	24	31.2	57	43	Pharmaceutical
	Placebo		121	24	31.6	56	40	Pharmaceutical
Pratley (2010) [39]	Liraglutide 1.2mg	Croatia, Germany, Ireland, Italy, Netherlands, Romania, Serbia, Slovakia, Slovenia, Spain, UK	221	6	32.6	55.9	48	Non- Pharmaceutical
	Liraglutide 1.8mg		218	6	33.1	55	48	Non- Pharmaceutical
	Sitagliptin 100mg		219	6	32.6	55	45	Non- Pharmaceutical
Russell Jones (2009) [45]	Liraglutide 1.8mg	USA, Argentina, Belgium, Brazil, UK, Canada, France, Germany, Hungary, India, Israel, Italy, Republic of Korea, Mexico, Poland, Puerto Rico, Romania, Slovakia, South Africa, Spain, Turkey	230	6	30.4	57.6	43	Pharmaceutical
	Placebo		114	6	31.3	57.5	51	Pharmaceutical
	Insulin Glargine		232	6	30.3	57.5	40	Pharmaceutical
Russell-Jones (2012) [40]	Exenatide 2mg/week	USA, Argentina, Belgium, Brazil, UK, Canada, France, Germany, Hungary, India, Israel, Italy, Republic of Korea, Mexico, Poland, Puerto Rico, Romania, Slovakia, South Africa, Spain, Turkey	248	6	31.4	51	44	Non- Pharmaceutical
	Metformin 2000mg		246	6	30.7	54	37.4	Non- Pharmaceutical
	Pioglitazone 45mg		163	6	31.1	55	40.5	Non- Pharmaceutical
	Sitagliptin 100mg		163	6	31.8	52	42.3	Non- Pharmaceutical
Yuan (2012) [41]	Exenatide 20μg	China	33	6	30.6	58.5	49	Non- Pharmaceutical
	Metformin 1500mg		26	6	29.3	56.8	54	Non- Pharmaceutical
Zinman (2009) [42]	Liraglutide 1.2mg	USA, Canada	178	6	33.2	55	43	Pharmaceutical
	Liraglutide 1.8mg		178	6	33.5	55	49	Pharmaceutical
	Placebo		177	6	33.5	55	49	Pharmaceutical

### Quality assessment

Out of the 27 studies that were eligible for inclusion, 93% provided the details of the randomisation process (S1 Table). Only 19% of trials gave information on the allocation process and 19% also recorded the double blinding of the study with adequate information. For the flow of participants, 85% provided full details of participants.

### Evidence Synthesis

From the pair-wise analysis, exenatide 20μg, liraglutide 1.2mg and liraglutide 1.8mg all had mean weight losses significantly greater than placebo of 1.32kg (95% CI 0.21kg, 2.43kg; n = 7 studies), 1.31kg (95% CI 0.77kg, 1.85kg; n = 2 studies) and 1.70kg (95% CI 0.80kg, 2.60kg; n = 3 studies) respectively. The difference in weight loss between exenatide 20μg and exenatide 2mg/week was small and non-significant: 0.05kg (95% CI -1.17kg, 1.27kg; n = 3 studies) as was the weight loss between liraglutide 1.2mg and liraglutide 1.8mg: 0.43kg (95% CI -0.1kg, 1.0kg; n = 3 studies). Liraglutide 1.8mg had a slightly greater weight loss that exenatide 20μg, 0.3kg (95% CI -0.60kg, 1.2kg; n = studies) which was once again not significant. There were no direct comparisons between exenatide 2mg/week and placebo, liraglutide 1.2mg or liraglutide 1.8mg. Neither was there a direct comparisons between exenatide 20μg and liraglutide 1.2mg.

In the mixed treatment comparison, the three GLP-1 receptor agonist treatments all showed a greater mean weight loss than placebo (exenatide 20μg: -1.37kg (95% Credible Interval (CrI) -2.22kg, -0.52kg); exenatide 2mg/week: -1.62kg (95% CrI -2.95kg, -0.30kg); liraglutide 1.2mg: -01.01kg (95% CrI -2.41kg, 0.38kg); liraglutide 1.8mg -1.51kg(95% CrI -2.67kg, -0.37kg). The weight loss seen with liraglutide 1.2mg was non-significant, however all the others saw significant weight loss and ranked as the best treatments (Table 2). Differences in average weight loss between the GLP-1 receptor agonists were small and non-significant (liraglutide 1.2mg vs exenatide 20μg: -0.36kg (95% CrI -1.83kg, 1.10kg); liraglutide 1.2mg vs exenatide 2mg/week: -0.61kg (95%CrI -2.32kg, 1.06kg); liraglutide 1.2mg vs liraglutide 1.8mg: -0.50kg (95% CrI -0.80kg, 1.78kg); exenatide 20μg vs exenatide 2mg/week: -0.25kg, (95% CrI -0.78kg, 1.28kg); exenatide 20μg vs liraglutide 1.8mg: -0.15kg (95% CrI -1.08kg, 1.38kg); exenatide 2mg/week vs liraglutide 1.8mg: -0.11kg (95%CrI -1.63kg, 1.41kg)).

**Table 2:** Results of Mixed Treatment Comparison.

Treatment	Rank (95% CrI)	Probability best treatment (%)	Mean weight change compared with placebo (kg) (95% CrI)
Exenatide 2mg	3 (1, 6)	26.04	-1.62 (-2.95, -0.30)
Liraglutide 1.8mg	3 (1, 6)	19.76	-1.51 (-2.67, -0.37)
Exenatide 20μg	4 (1, 6)	6.45	-1.37 (-2.22, -0.52)
Liraglutide 1.2mg	5 (1, 8)	5.83	-1.01 (-2.41, 0.38)
Sitagliptin	5 (1, 10)	14.72	-0.88 (-3.31, 1.63)
Metformin	5 (1, 9)	8.54	-0.95 (-2.75, 0.86)
Insulin Detemir + Liraglutide 1.8 mg	6 (1, 11)	14.17	-0.71 (-3.33, 1.99)
Placebo	8 (5, 10)	0.02	—
Exenatide 20μg + Rosiglitazone	8 (1, 13)	4.45	0.25 (-2.54, 3.00)
Insulin Detemir	11 (8, 15)	0.01	1.85 (-0.06, 3.79)
Glimepiride	11 (9, 15)	0.00	1.93 (0.30, 3.54)
Pioglitazone	11 (9, 15)	0.00	1.99 (0.18, 3.84)
Rosiglitazone	13 (9, 16)	0.02	2.94 (0.17, 5.69)
Insulin Glargine	14 (11, 16)	0.00	3.12 (1.47, 4.78)
Glibenclamide	15 (10, 16)	0.01	3.83 (1.25, 6.40)
Biphasic Insulin Aspart	15 (11, 16)	0.00	4.04 (1.49, 6.60)

### Model diagnostics

The I^2^ statistic was larger than 75% for four comparisons in the analysis (exenatide 20μg vs placebo, insulin glargine and exenatide 2mg/week, as well as pioglitazone vs exenatide 2mg/week and sitagliptin). Due to a limited number of direct comparisons, the I^2^ statistic could not be calculated for several comparisons. The mixed treatment comparison estimated the between trial variance to be 1.28 (95% CrI 0.58, 2.56), which supports the heterogeneity in the pair-wise meta-analysis.

There were seven inconsistencies where the mixed treatment comparison and pair-wise meta-analysis results differed substantially; namely, exenatide 2mg/week vs insulin glargine, exenatide 2mg/week vs metformin, exenatide 20μg vs metformin, liraglutide 1.2mg vs pioglitazone, liraglutide 1.8mg vs pioglitazone, pioglitazone vs insulin detemir and pioglitazone vs metformin. A contour enhanced funnel plot of the comparison between placebo and exenatide 20μg was produced as this was the only comparison for which more than five studies were included (S1 Fig). There is some indication that studies in the area of statistical non-significance may be missing systematically, thus publication bias may be present.

The model had a good level of fit as the total residual deviance values (61.5) was close to the number of unconstrained data points (60). The between trial variance for the analysis was 1.28 (95% CrI 0.59, 2.56) which does suggest there is heterogeneity present.

## Discussion

A pair-wise meta-analysis and mixed treatment comparison found that the GLP-1 receptor agonist therapies considered resulted in a reduction in body weight; these reductions were only significant in some analyses. Differences in average weight loss between the GLP-1 receptor agonists were small and non-significant

These findings add to evidence that GLP-1 receptor agonists may have weight loss benefits in patients with type 2 diabetes. These weight loss effects have been shown previously [17], and it is believed that GLP-1 receptor agonists affect weight loss through their effects on appetite and satiety [18]. GLP-1 is a gut hormone secreted from the lower intestinal endocrine L-cells following the ingestion of food. It has a number of functions which include augmenting insulin’s response to glucose, slowing gastric emptying, suppressing the secretion of glucagon and thus hepatic glucose output, and increasing satiety. Our findings further suggest that weight loss effects may differ between the GLP-1 receptor agonists, possibly due to the different homologies of the drugs and the different frequencies with which they are administered. Interestingly the level of weight loss seen was consistent across BMI (S2 Fig).

In previous systematic reviews and meta-analyses of GLP-1 receptor agonist therapies, weight change is usually not the primary outcome and so is not well reported. Instead, the meta-analyses primarily look at the effect of these therapies on other outcome measures, such as HbA1c, on which they have a positive effect [19]. There has only been one meta-analysis of weight loss for GLP-1 receptor agonist therapies (exenatide or liraglutide at a clinically relevant dose), which found that they were associated with a greater weight loss than control intervention (-2.9kg; 95% CI -3.6kg, -2.2kg) [6]. The control intervention was a pooled group of placebo and other treatments. This weight loss was also seen in the subgroup of participants with type 2 diabetes (-2.8kg; 95% CI -3.4kg, -2.3kg). Other studies also support the results of this mixed treatment comparison analysis, but do not show the difference in weight loss between the GLP-1 receptor agonist treatments. These include a pooled analysis of phase three trials from the Liraglutide Effect and Action in Diabetes (LEAD) program, which found a statistically significant reduction in body weight for liraglutide 1.2mg versus placebo across different age groups [20], and a pooled analysis looking at the efficacy and tolerability of exenatide once weekly, which found that it was significantly associated with reduced body weight, taking into account the baseline glucose lowering therapy that the patient was already receiving [21]. The average weight loss observed in the current analysis was lower than in the previous meta-analysis at -1.01kg for liraglutide 1.2mg, -1.51kg for liraglutide 1.8mg, -1.37kg for exenatide 20μg and -1.62kg for exenatide 2mg/week. Though these amounts are small, they are likely to confer some clinical benefit since it has been shown that, on average, each 1kg of weight loss is associated with 3–4 months additional survival in people with type 2 diabetes [22]. Furthermore, the other anti-diabetes medications considered were all associated with weight gain on average, albeit not always significantly so, highlighting the weight loss benefits of the GLP-1 receptor agonists, and it might be that longer treatment durations would have resulted in greater weight loss.

The previous pair-wise review included studies of people with type 2 diabetes and those without type 2 diabetes [6], three studies were conducted in those without type 2 diabetes. A comparable weight loss was seen to those conducted in people with type 2 diabetes. We did not include studies conducted in people without type 2 diabetes. Given the limited evidence base for those without diabetes future research should focus on whether GLP-1 receptor agonists can be used for weight loss in those who are free from type 2 diabetes but overweight or obese.

The risk of bias was assessed in the trials included in the analysis. Most studies were able to account for the participants who dropped out with detailed information, and there was adequate reporting on randomisation. The percentage of trials reporting double blinding was quite small (19%), suggesting bias might have been introduced. The lack of blinding is probably a result of the interventions being delivered differently; GLP-1 receptor agonist therapies and insulin are injectables and all other therapies are administered orally. The outcome is weight which can be recorded accurately and objectively at any stage of the trial, and should not be influenced by an investigator, therefore the effect of non-blinding is likely to be minimal. Publication bias may have been present with non-significant studies appearing to be less likely to be published. This might mean that the published literature is biased towards showing a weight loss effect of GLP-1 receptor agonists.

The mixed treatment comparison allowed direct and indirect evidence to be included in a single evidence synthesis while preserving the randomisation. This also allowed the GLP-1 therapies to be analysed separately instead of combined, meaning that no assumption was made about the similarity of the behaviour of the GLP-1 receptor agonist therapies. The background treatment was ignored, making the assumption that any treatment given alongside the primary intervention or the control did not affect the behaviour of the intervention or alter the weight change seen. This was done to make the trials more comparable, to simplify the treatments given and to limit the number of interventions considered. It is seen that different classes of interventions give different weight changes. This could suggest that this assumption may not be valid, and that the weight change seen may be exaggerated or limited if given with a background treatment which produces a weight loss or gain. This limits the analysis as it assumes that all the background treatments behave in the same way and it may have been more useful to stratify the pharmaceutical interventions into the different types of anti-diabetic medication given to account for their different effects on weight.

The mixed treatment comparison analyses allows treatments to ranked in terms of the probability that they are best treatment. Exenatide 2mg/week appeared to be the best treatment out of the GLP-1 receptor agonists as it was ranked higher than exenatide 20μg and liraglutide 1.8mg, though the differences in weight loss between the treatments were small and non-significant. With the exception of liraglutide 1.2mg the GLP-1 receptor agonist therapies considered ranked higher than the comparator treatments. These results should be interpreted with caution though as recent work by Kibret et al [46] showed that the rank probability is sensitive to various factors including unequal numbers of studies in the comparisons and whether network contains loops, both of which appear in our network.

A major strength of this work is that the systematic review conducted was extensive, using all licenced GLP-1 receptor agonist therapies, so it was unlikely that trials of interest were missed. Due to there being no eligible studies using lixisenatide at the time of the review, only the effects of liraglutide and exenatide were considered. The GLP-1 therapies were considered at clinically relevant doses which makes the research relevant to clinical practice, even though one dose liraglutide 1.8mg is not recommended for use in the UK [16]. A limitation was that full text searching and data extraction were done by three individuals (JP, DB and LG). However discussions were had so that the information included was consistent to avoid bias. GLP-1 receptor agonist therapies are associated with side effects including nausea, diarrhoea, headaches, dizziness and vomiting. These side effects may escalate the amount of weight loss, and so further investigation needs to be conducted to look at the adverse events to see whether there are unacceptable levels of adverse events, which outweigh the weight loss benefits in participants. The review was conducted following the guidelines of the PRISMA statement (S2 File). Further work may also be needed to attempt to explain heterogeneity seen by adding study level covariates into the model in order to adjust for potentially unevenly distributed covariates between trials [15].

In conclusion, the analyses undertaken provide evidence that treatment with GLP-1 receptor agonist therapies for 6 months was associated with a reduction in body weight in participants who are overweight or obese with type 2 diabetes. There is no clear evidence as to which GLP-1 receptor agonist leads to the greatest weight loss, although our analyses may suggest that exenatide is superior to liraglutide, although more data is required to confirm this. Further investigation needs to be conducted to determine the effects of GLP-1 receptor agonist therapy in people without diabetes who are overweight or obese.

## Supporting Information

S1 Fig Contour enhanced funnel plot. (EPS)

S2 Fig Scatterplot of weight loss and BMI. (EPS)

S1 File Example of search strategy used in Medline. (PDF)

S2 File Prisma Statement. (PDF)

S1 Table Risk of bias in the included studies. (PDF)
