# Supplementary material for: The Effect of Glucagon-Like Peptide 1 Receptor Agonists on Weight Loss in Type 2 Diabetes: A Systematic Review and Mixed Treatment Comparison Meta-Analysis
Source: PLoS One. 2015 Jun 29;10(6):e0126769. doi: 10.1371/journal.pone.0126769 (PMC4487255; doi:10.1371/journal.pone.0126769)
Supplement: S1 File — (PDF) [file pone.0126769.s003.pdf]

File S1. Example of search strategy used in Medline

Database: Ovid MEDLINE(R) In-Process & Other Non-Indexed Citations and Ovid MEDLINE(R) <1946 to Present>
Search Strategy:
----

1 Glucagon-Like Peptide 1/ or "Glucagon like peptide 1".mp.
2 (glucagon* adj2 peptide).mp.
3 GLP*1.mp.
4 Exenatide.mp.
5 Liraglutide.mp.
6 Albiglutide.mp.
7 Taspoglutide.mp.
8 Lixisenatide.mp.
9 1 or 2 or 3 or 4 or 5 or 6 or 7 or 8
10 Obesity/ or Obesity.mp.
11 overweight.mp. or Overweight/ or Body Weight/
12 obese.mp.
13 Body Mass Index/ or "BMI".mp.
14 "Body mass index".mp.
15 weight.mp.
16 (weight adj2 loss).mp.
17 Weight Loss/
18 10 or 11 or 12 or 13 or 14 or 15 or 16 or 17
19 (randomised adj2 trial*).mp.
20 (randomized adj2 trial*).mp.
21 "RCT".mp.
22 Randomized Controlled Trials as Topic/
23 19 or 20 or 21 or 22
24 9 and 18 and 23
