# Supplementary material for: The Effect of Glucagon-Like Peptide 1 Receptor Agonists on Weight Loss in Type 2 Diabetes: A Systematic Review and Mixed Treatment Comparison Meta-Analysis
Source: PLoS One. 2015 Jun 29;10(6):e0126769. doi: 10.1371/journal.pone.0126769 (PMC4487255; doi:10.1371/journal.pone.0126769)
Supplement: S1 Table — (PDF) [file pone.0126769.s005.pdf]

Table S1. Risk of bias in the included studies

	Randomisation	Allocation Concealment	Double Blinding	Flow of Participants
Apovian (2010)	2	0	1	2
Bergenstal (2009)	2	0	0	1
Bergenstal (2010)	2	1	2	2
Blevins (2011)	2	0	0	2
Buse (2011)	2	1	2	2
Buse (2004)	2	0	2	2
Buse (2009)	2	0	0	2
Davies (2013)	2	0	0	2
Davies (2009)	2	0	0	2
DeFronzo (2010)	2	0	0	2
DeFronzo (2005)	2	0	2	2
Derosa (2010)	2	0	1	1
Derosa (2011)	2	0	0	2
DeVries (2011)	1	0	0	2
Diamant (2010)	2	0	0	2
Drucker (2008)	2	0	0	2
Heine (2005)	2	0	0	2
Ji (2013)	2	0	0	2
Kadowaki (2011)	2	1	1	2
Kendall (2005)	2	0	0	2
Liutkus (2010)	2	0	1	2
Nauck (2009)	2	0	0	2
Pratley (2010)	2	1	1	2
Russell-Jones (2009)	2	1	2	2
Russell-Jones (2012)	2	0	1	2
Yuan (2012)	1	0	0	1
Zinman (2009)	2	0	1	1

Key:

Randomisation 0=not randomised, 1=randomised, no reported methods, 2=randomised and method reported;

Allocation Concealment 0=not reported, 1=concealment reported;

Double Blinding 0=single blind, 1=double blind, no detail, 2=double blind, detailed method;

Flow of Participants 0=not given, 1=brief mention of drop outs, 2=detailed description of drop outs.
